# Supplementary figures and images for: Fine-scale differentiation between Bacillus anthracis and Bacillus cereus group signatures in metagenome shotgun data
Source: PeerJ. 2018 Aug 22;6:e5515. doi: 10.7717/peerj.5515 (PMC6109372; doi:10.7717/peerj.5515)

**A**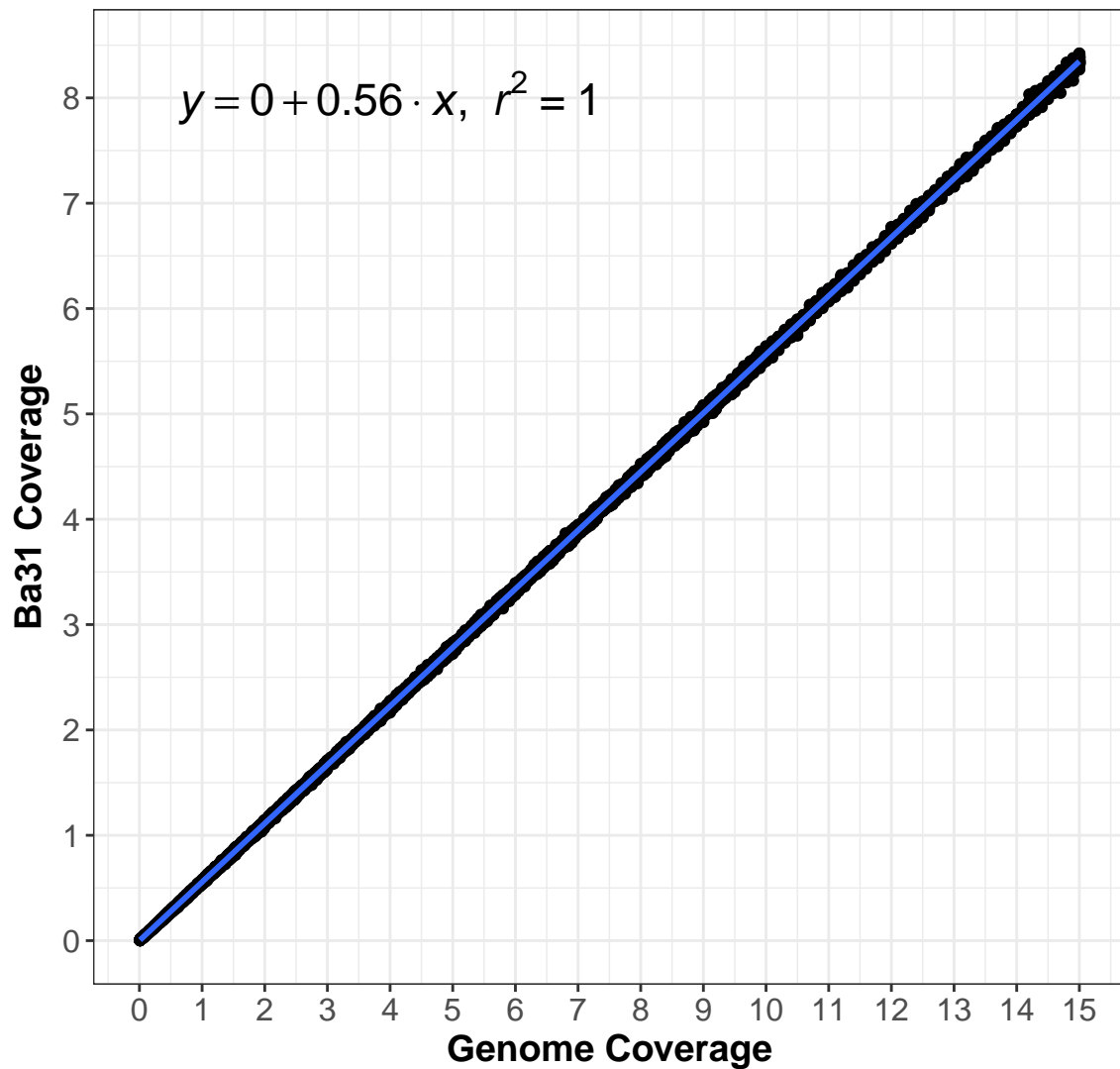**B**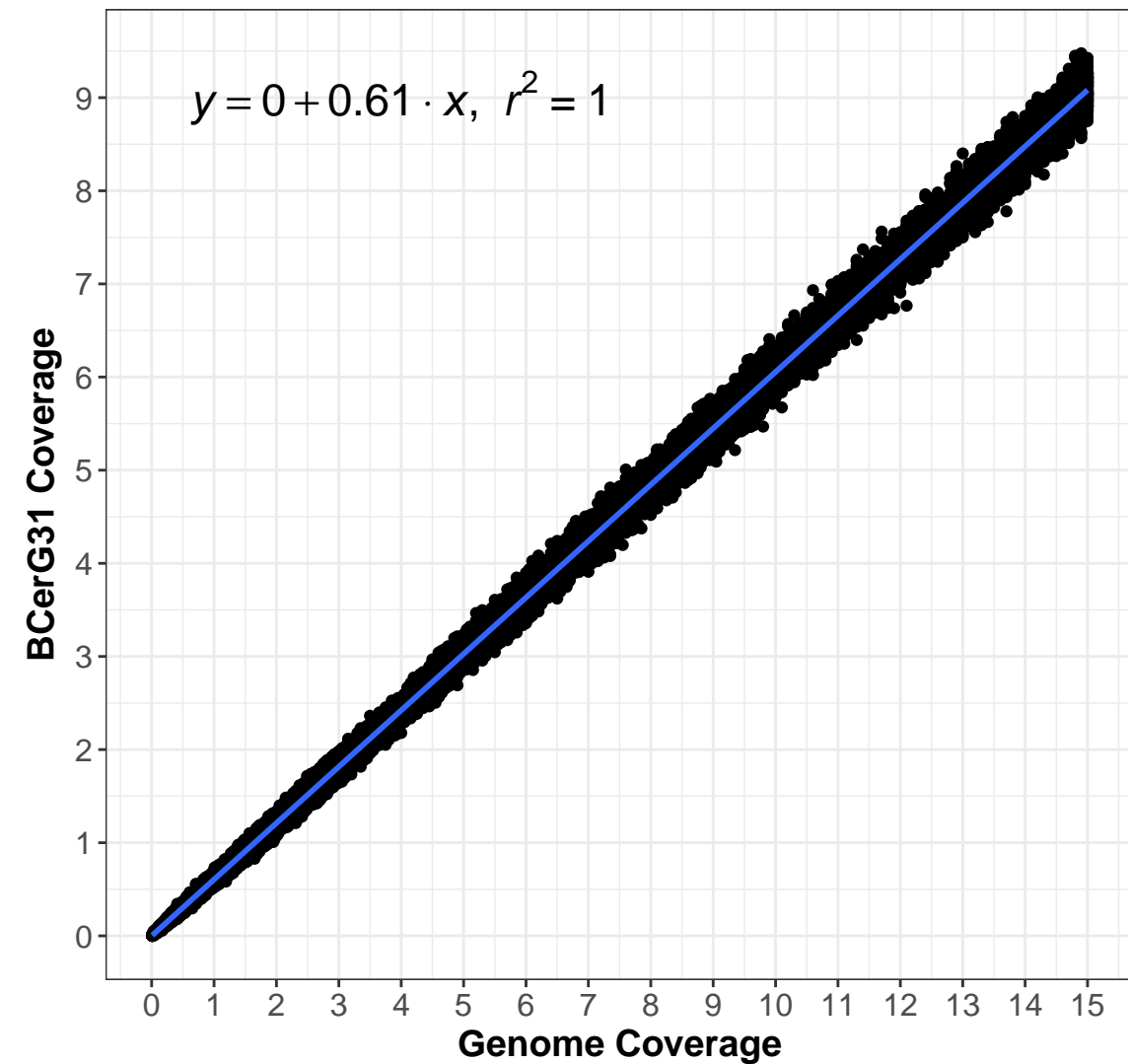

Supplement: Figure S1 — We created synthetic FASTQ files of B. anthracis (A) and BCerG (B) at different genome coverages and counted Ba31 and BCerG31 k-mers. A linear model with an intercept of 0 is displayed in each case. [file peerj-06-5515-s001.pdf]

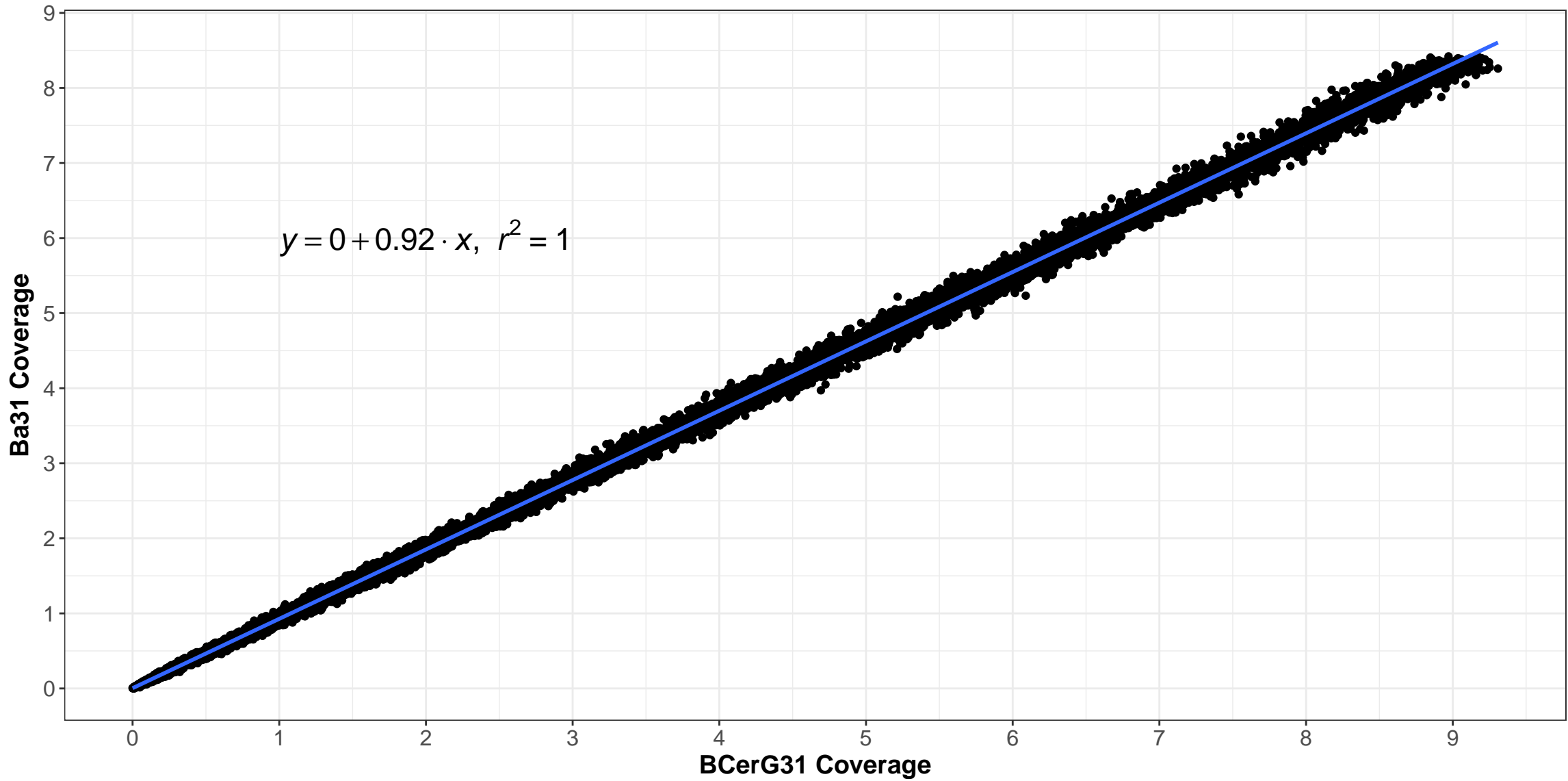

Supplement: Figure S2 — We created synthetic B. anthracis FASTQ files at different genome coverages and counted BCerG31 and Ba31 k-mers. A linear model with an intercept of 0 is displayed. [file peerj-06-5515-s002.pdf]

Ba31 Coverage

$$y = 0 + 0.00027 \cdot x, \quad r^2 = 0.597$$

0.0000

0.0025

0.0050

0.0075

0

1

2

3

4

5

6

7

8

9

BCerG31 Coverage

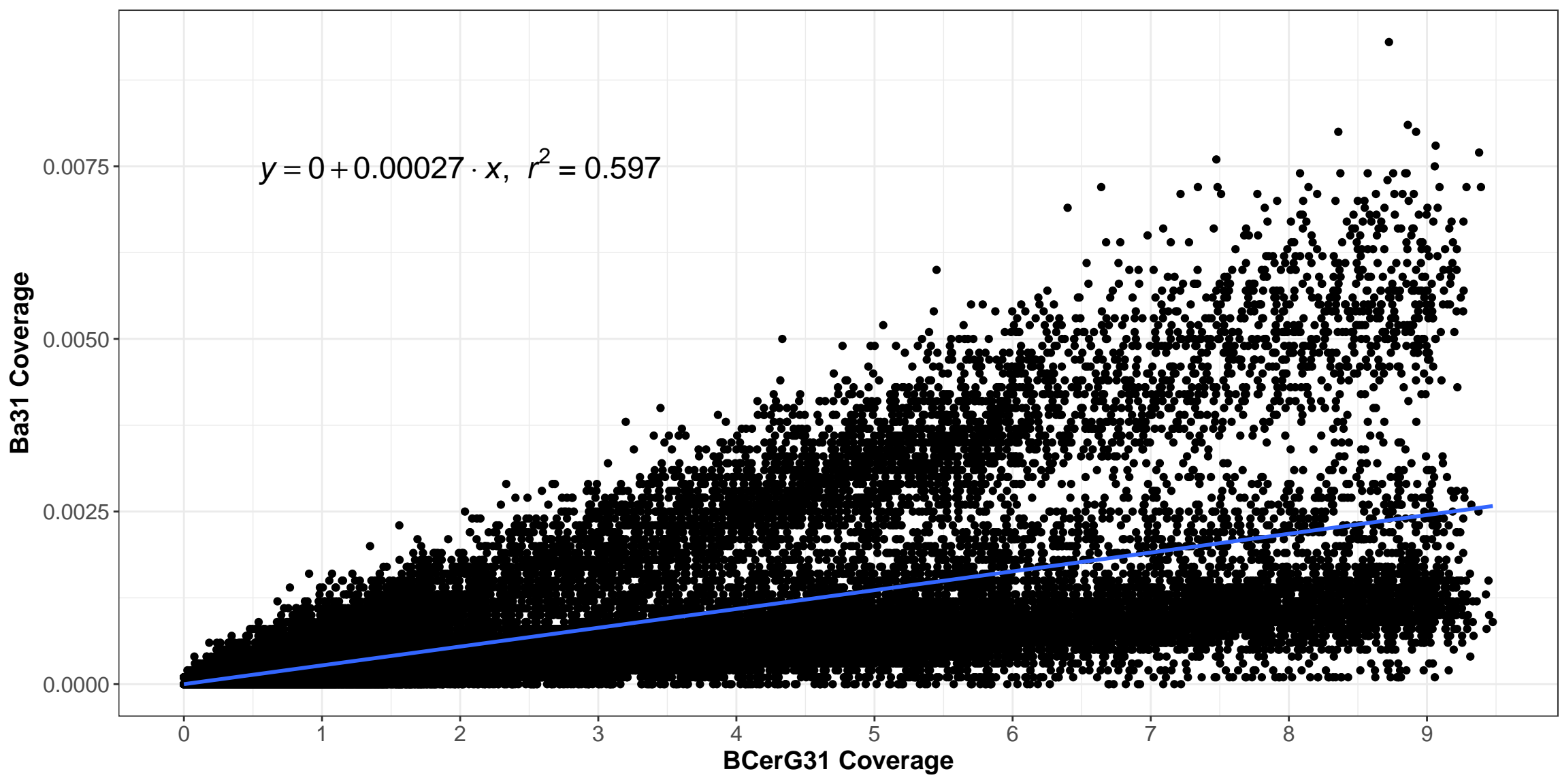

Supplement: Figure S3 — We created synthetic non-B. anthracis FASTQ files at different genome coverages and counted BCerG31 and Ba31 k-mers. A linear model with an intercept of 0 is displayed. [file peerj-06-5515-s003.pdf]

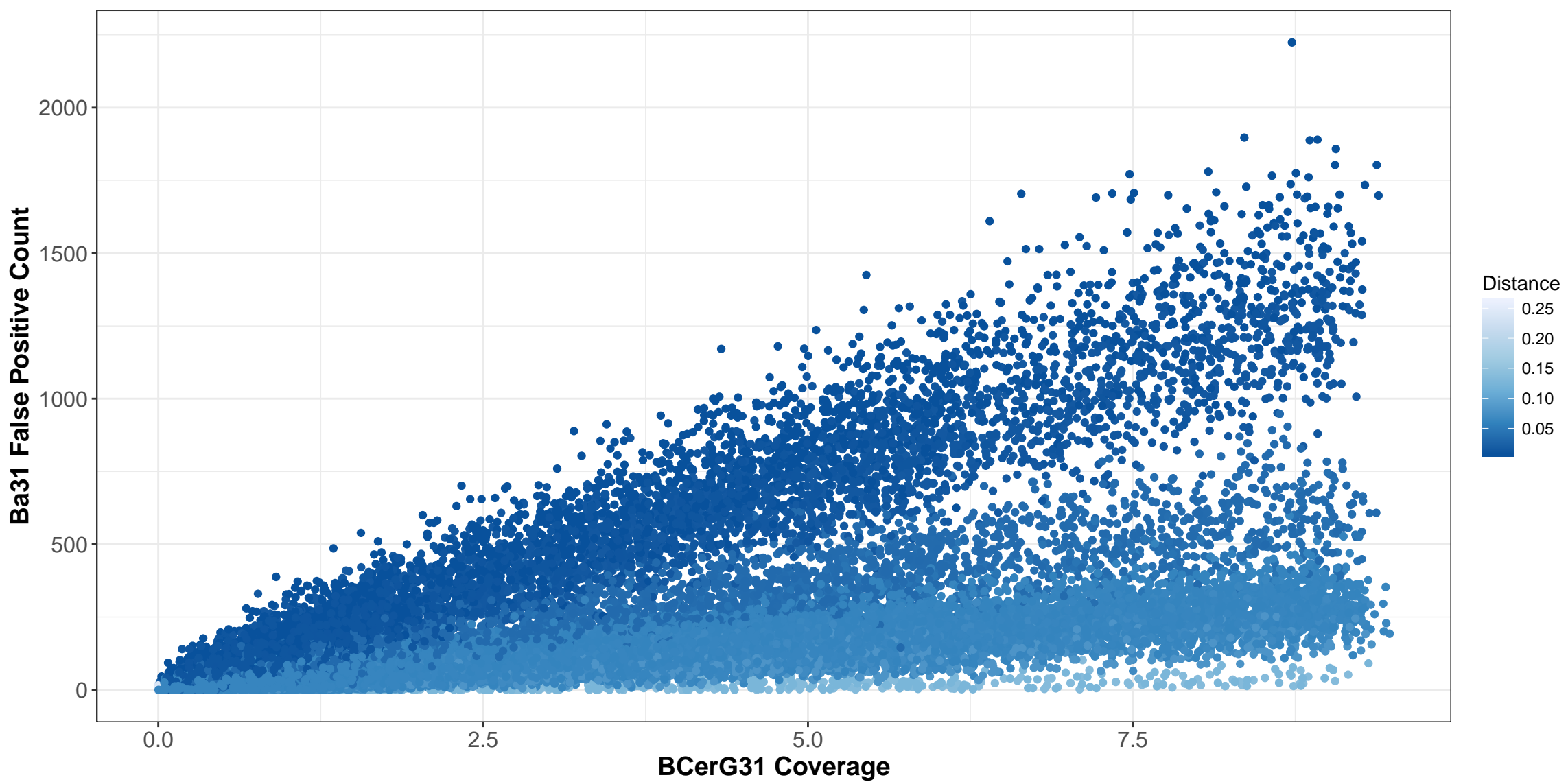

Supplement: Figure S4 — Synthetic FASTQ files for all BCerG genomes shown in Fig. 2 were created and the counts of Ba31 false positive k-mers were plotted against BCerG k-mer coverage. Dots are colored by the Mash distance (Ondov et al., 2016) from the B. anthracis str. Ames (NC_003997) genome. [file peerj-06-5515-s004.pdf]

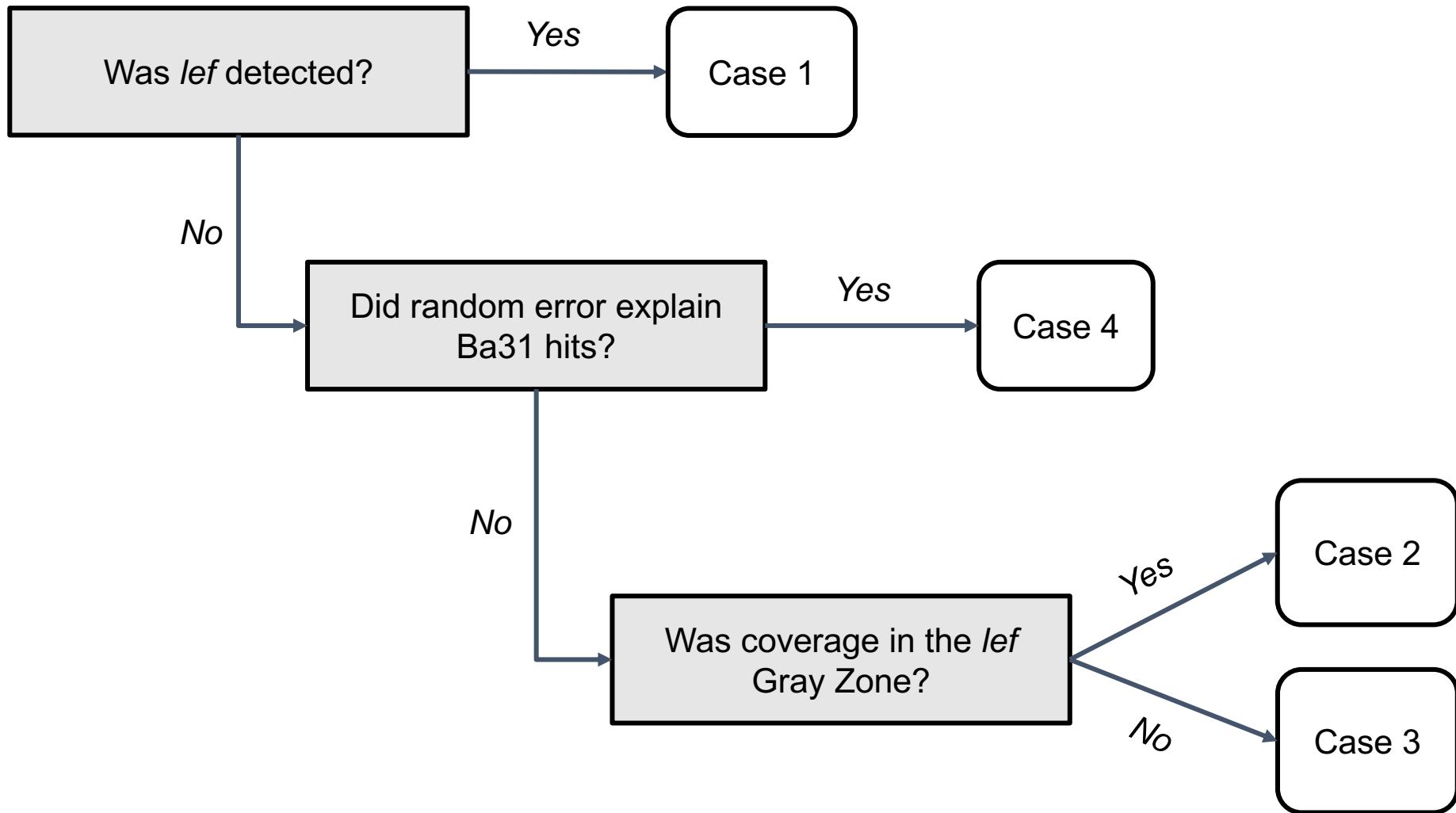

Supplement: Figure S5 — This flowchart presents a visual representation of Table 3. [file peerj-06-5515-s005.pdf]

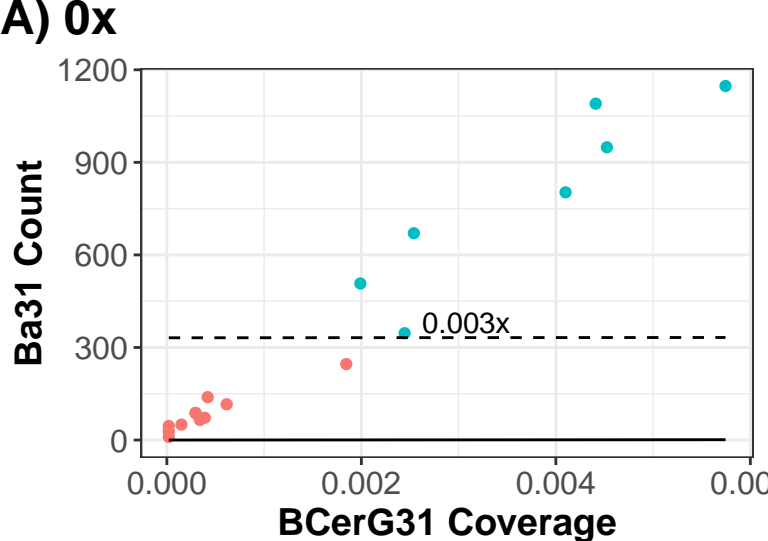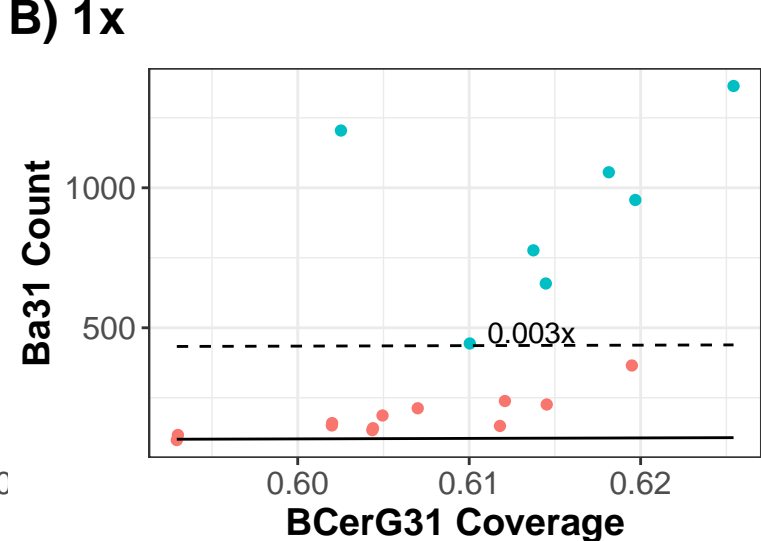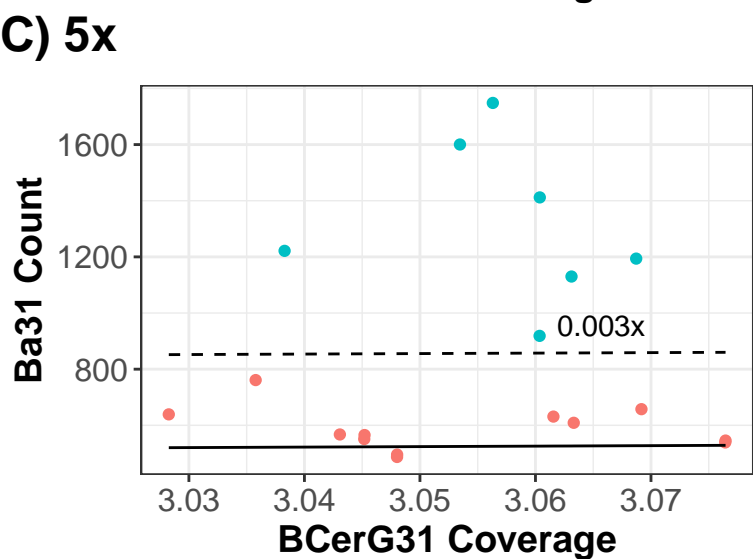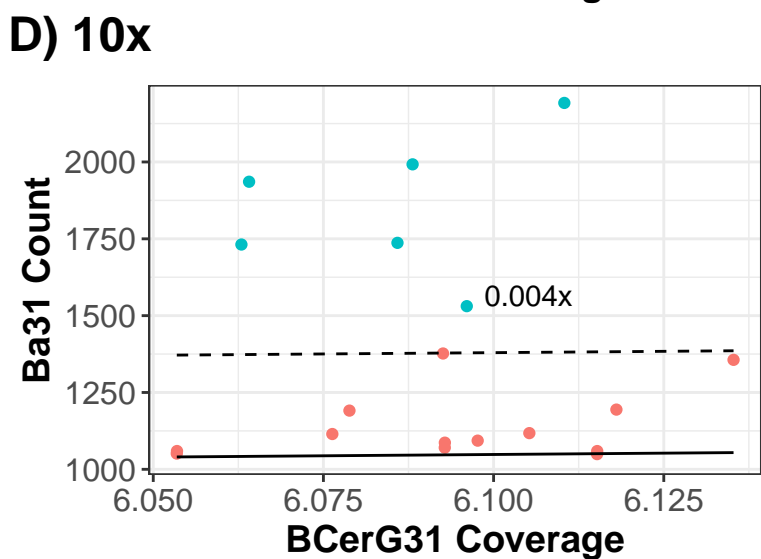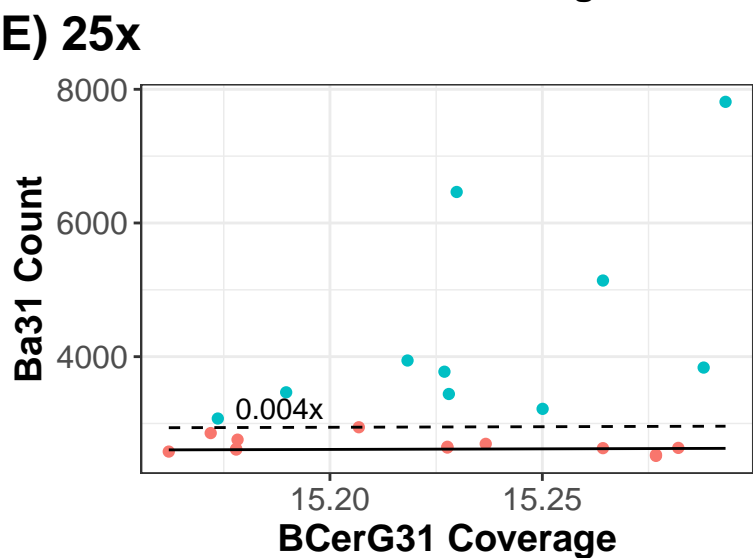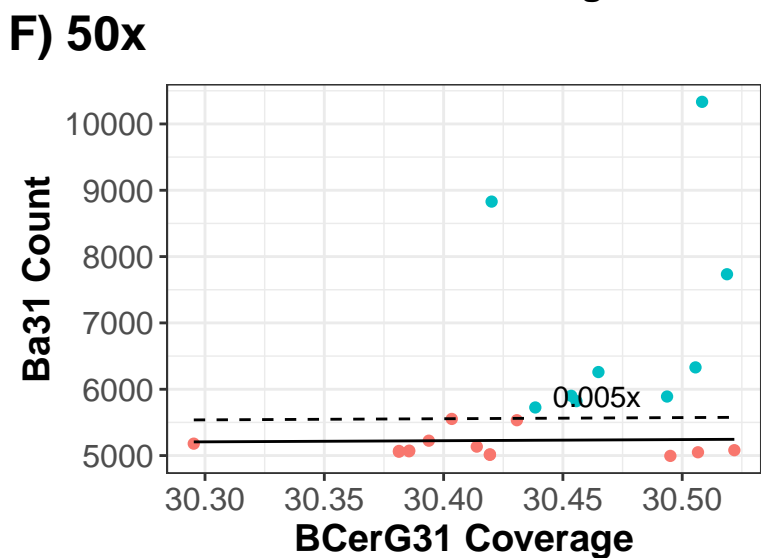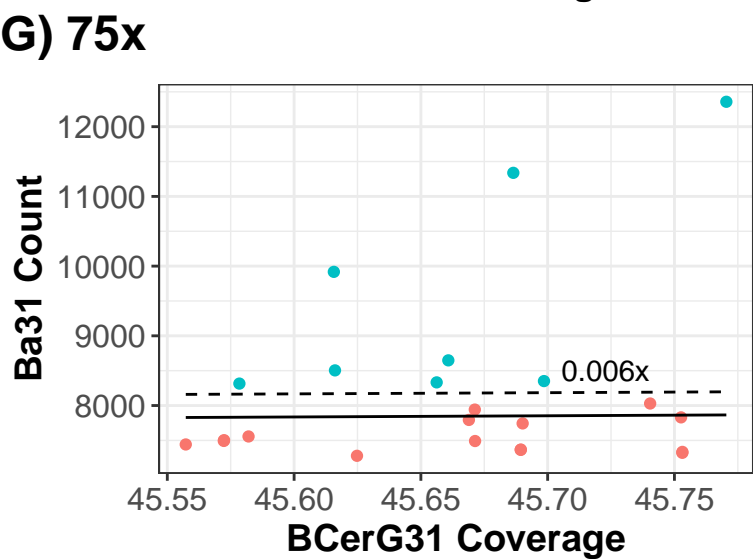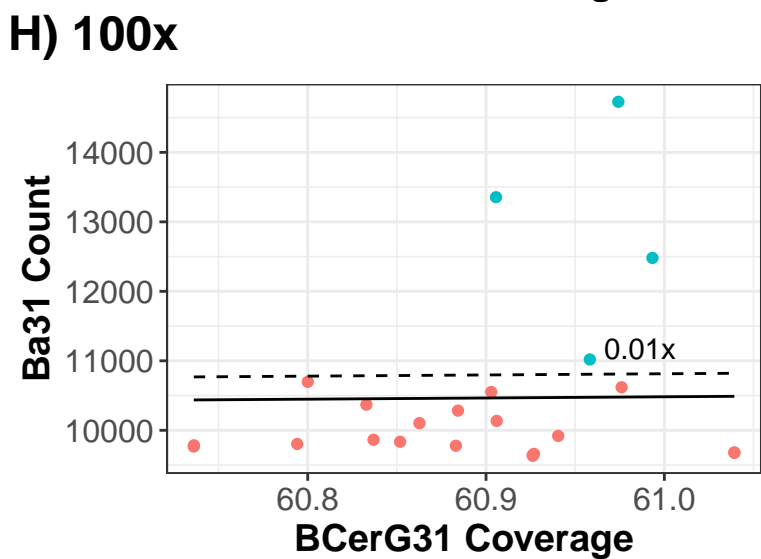

Ba31 Detectable FALSE TRUE

Supplement: Figure S6 — We created artificial mixtures of B. anthracis and B. cereus to determine the limit of detection for B. anthracis k-mers (Ba31). Each panel represents a different coverage of B. cereus and the points are the different B. anthracis coverages. The points are colored red if Ba31 matches could not be differentiated from sequencing errors. The error model is indicated by the solid line and the 99% prediction interval by the dashed line. The first B. anthracis coverage value that exceeded the error model is determined as the limit of detection of Ba31. [file peerj-06-5515-s006.pdf]
